# Decreased levels of genuine large free hCG alpha in men presenting with abnormal semen analysis

**DOI:** 10.1186/1477-7827-9-114

**Published:** 2011-08-12

**Authors:** Christoph Zenzmaier, Regine Gerth, Matthias Gruschwitz, Herbert Lindner, Eugen Plas, Peter Berger

**Affiliations:** 1 Institute for Biomedical Aging Research, Austrian Academy of Sciences, Rennweg 10, 6020 Innsbruck, Austria; 2 Department of Dermatology, University of Erlangen-Nuremberg, Hartmannstraße 14, 91052 Erlangen, Germany; 3 Division of Clinical Biochemistry and Protein Micro-Analysis Facility, Medical University Innsbruck, Fritz-Pregl-Str. 3, 6020 Innsbruck, Austria; 4 Department of Urology and Ludwig Boltzmann Institute for Urology and Andrology, Hospital Hietzing, Wolkersbergenstraße 1, 1130 Vienna, Austria

## Abstract

**Background:**

The pregnancy hormone human chorionic gonadotropin (hCG) and its free subunits (hCG alpha, hCG beta) are produced in the male reproductive tract and found in high concentrations in seminal fluid, in particular hCG alpha. This study aimed to elucidate changes in peptide hormone profiles in patients showing abnormal semen analyses and to determine the genuineness of the highly abundant hCG alpha.

**Methods:**

Seminal plasma was obtained from 45 male patients undergoing semen analysis during infertility workups. Comprehensive peptide hormone profiles were established by a panel of immunofluorometric assays for hCG, hCG alpha, hCG beta and its metabolite hCG beta core fragment, placental lactogen, growth hormone and prolactin in seminal plasma of patients with abnormal semen analysis results (n = 29) versus normozoospermic men (n = 16). The molecular identity of large hyperglycosylated hCG alpha was analyzed by mass-spectrometry and selective deglycosylation.

**Results:**

hCG alpha levels were found to be significantly lower in men with impaired semen quality (1346 +/- 191 vs. 2753 +/- 533 ng/ml, *P *= 0.022). Moreover, patients with reduced sperm count had reduced intact hCG levels compared with normozoospermic men (0.097 +/- 0.022 vs. 0.203 +/- 0.040 ng/ml, *P *= 0.028). Using mass-spectrometry, the biochemical identity of hCG alpha purified from seminal plasma was verified. Under non-reducing conditions in SDS-PAGE, hCG alpha isolated from seminal plasma migrated in a manner comparable with large free hCG alpha with an apparent molecular mass (Mr, app) of 24 kDa, while hCG alpha dissociated from pregnancy-derived holo-hCG migrated at approximately 22 kDa. After deglycosylation with PNGase F under denaturing conditions, all hCG alpha variants showed an Mr, app of 15 kDa, indicating identical amino acid backbones.

**Conclusions:**

The findings indicate a pathophysiological relevance of hCG, particularly its free alpha subunit, in spermatogenesis. The alternative glycosylation pattern on the free large hCG alpha in seminal plasma might reflect a modified function of this subunit in the male reproductive tract.

## Background

Male fertility abnormalities are diagnosed by physical examination, endocrine parameters and assessment of semen quality. Endocrine analyses include hormones of the pituitary testicular axis, i.e. pituitary-derived gonadotropins luteinizing hormone (LH), follicle stimulating hormone (FSH), total testosterone (TT) and free/bioavailable testosterone. Other hormones, such as prolactin (hPRL), estrogen, or stress hormones, are also important parameters of the male fertility workup.

**Figure 1:**
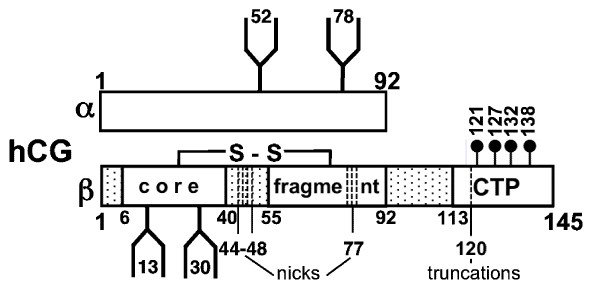
**Schematic depiction of the molecular structures of the cystine knot growth factor hCG and of hCG-variants**. hCG consists of an α-subunit (hCGα) with 92 amino acids (aa) in length non-covalently linked to the hCGβ-subunit (145 aa). Nicked hCG (hCGn) and nicked hCGβ (hCGβn) contain nicks in the region of aa hCGβ44-48 and rarely around aa 77 of hCGβ. The most important metabolic product of hCG is the hCGβ core fragment (hCGβcf), which is composed of two peptides encompassing aa hCGβ6-40 and 55 to 92 covalently linked. The hCG-unique carboxyl-terminal peptide (CTP) is clipped and truncated hCG and hCGβ variants are generated (-CTPhCG and -CTPhCGβ). There are a multitude of glycosylation variants involving both N-glycosylated (hCGα aa 52 & 78, hCGβ aa 13 & 30) and O-glycosylated carbohydrate antennae (hCGβ aa Ser 121, 127, 132, 138) [41,42].

Apart from serum hormone levels, investigators sought molecular markers of spermatogenesis in seminal plasma. Such markers were anti-Mullerian hormone, inhibin B [1], and transferrin. Although a few attempts have been made to correlate human chorionic gonadotropin (hCG) and hCG-like molecules to spermatogenesis [2-9], the molecular heterogeneity of molecular hCG species in various body fluids, the lack of appropriate standards of hCG-variants, undefined molecular recognition profiles of immunoassay analyses and interpretation of marker profiles has, until recently, been too poor to do so [10] (the molecular structures of hCG and its variants are schematically depicted in Figure 1). The International Society of Oncodevelopmental Biology and Medicine (ISOBM) has initiated and completed, as part of its Tissue Differentiation (TD) program, the international TD-7 Workshop on antibodies to hCG and hCG-related molecules and recommended epitope combinations for 2-site immunoassays for the measurement and determination of members of the molecular hCG family [11]. With our selective panel of immunofluorometric assays (IFMAs) based on the reference monoclonal antibodies (mAbs) of the TD-7 Workshop [12], we previously investigated the abundance of hCG and hCG-like molecules in seminal plasma of healthy fertile men and found significant differences between the hCG marker profile and that of other hCG-containing physiological body fluids [13]. During pregnancy, the α/β-heterodimer hCG is present in vast molar excess in serum, as is hCGβ/hCGβcf (hCGβ core fragment) in urine, but in seminal plasma, the subunit hCGα was by far the most prominent marker of the hCG molecular family, with concentrations as high as that found during pregnancy in extraembryonic coelomic fluid, and approximately 10,000-fold higher than in normal male serum. hCGβ and holo-hCG concentrations were found to be 1000-fold and 10,000-fold less than hCGα, whereas hCGβcf levels were mostly undetectable in seminal plasma [13].

In the present study, the local profile of endocrine parameters, i.e. hCG-like substances and the family of protein hormones prolactin (hPRL), growth hormone (GH) and placental lactogen (PL), was analyzed in seminal plasma of men with abnormal semen analysis findings and compared to normozoospermic men to clarify a possible pathophysiological role of these hormones in spermatogenesis disorders. An important question was whether the highly abundant hCGα is genuine hCGα or a moderately-defined hCGα-like substance. Thus, the glycosylation pattern of hCGα, which has two N-glycosylation sites at Asn^52 ^and Asn^78 ^[14], was purified from seminal plasma and the isolated protein analyzed by mass-spectrometry.

## Methods

### Determination of serum hormone levels

All blood samples were obtained between 8 and 10 a.m. and were immediately transferred to the laboratory for further processing. Hormonal analyses included the determination of FSH, LH, TT and hPRL. FSH and LH analysis was performed using a standard two step immunoassay (Architect FSH, Abbott; ref. B7K750; Architect LH, Abbott; ref. 34-4522/R8). hPRL levels were determined by the two-step immunoassay Architect Prolactin (Abbott; ref. B7K760), TT levels by the Architect Testosterone Assay (Abbott; ref. B7K730). All assays were performed according to manufacturer's instructions.

### Semen samples

Human semen samples were collected after 4 days of ejaculatory continence from 45 male patients undergoing semen analysis as part of an infertility workup at the Andrology Division of the Department of Dermatology, Univ. of Erlangen-Nuremberg, Germany, between 1995 and 1997. Semen samples were obtained and processed as recommended in the WHO Laboratory Manual for the Examination of Human Semen and Semen-Cervical Mucus Interaction [15]. None revealed the presence of antisperm antibodies in their seminal plasma or sera as shown by the immunobead technique [16]. The seminal fluid remaining after routine testing was aspirated from the spermatozoal pellet and stored at -20°C until use. After thawing, the seminal fluids were centrifuged at 40,000 × g to remove residual debris and then assayed. All patients gave written informed consent.

### Semen analysis

Semen analysis was performed by determination of pH, liquefaction time, volume, total and progressive motility at 60 min after ejaculation, sperm/round cell/germ cell/leukocyte counts, and the total and specific head/midpiece/tail percentage of abnormal forms according to the WHO criteria of 1995. Morphology was determined on methanol-fixed (2.5 min) and giemsa-stained (10 min) sperm smears by light microscopy and by two independent observers by using the strict Kruger criteria. Viability of sperm was measured by eosin Y staining. The concentration of polymorphonuclear neutrophil white blood cells was measured by peroxidase staining.

Patients were grouped according to their semen analysis; abnormal semen results (n = 29): oligoasthenoteratozoospermia (OAT, n = 7), asthenoteratozoospermia (n = 2), asthenozoospermia (n = 3), teratozoospermia (n = 1), oligozoospermia (n = 2), cryptozoospermia (n = 8), azoospermia (n = 6), and normozoospermic men (n = 16).

### Time-resolved immunofluorometric assays

Generation and characterization of monoclonal Antibodies (mAbs) against intact hCG, hCGβ, hCGβcf, hCGα and FSH and GH, hPRL and PL has been described in detail [17-20]. The mAbs against hCG/hCG-variants were previously used as reference reagents in the international TD-7 Workshop on antibodies to hCG and hCG-related molecules [11]. Sensitive and specific IFMAs for hCG, free hCGα, free hCGβ and the hCGβcf, respectively, were developed on the basis of our panel of mAbs [21,22]. MAb pairs for each of the four IFMAs were selected on the basis of antigen specificity, epitope localization and compatibility [11,17,18,23-25].

IFMAs were performed as published previously [26,27]. Samples were diluted in 0.01 mol/l NaHCO_3 _containing 0.1% bovine serum albumin (BSA).

The National Institute for Biological Standards and Control (NIBSC; South Mimms, UK) kindly provided the International Standards (IS) for hCG IS75/537, hCGβ IS 75/551, hCGα IS 74/569. Highly purified hCGβcf was a gift by Drs. Klaus Mann and Rudy Hoermann (Essen, Germany).

Coating mAbs were coded as INN(sbruck)-hCG-45 (hCG+hCGn-assay), -68 (hCGβ-assay), -106 (hCGβcf-assay) and INN-hCG-72 (hCGα-assay). The detection mAbs (INN-hFSH-158 for the hCGα-assay were directed against epitope α3, presumably located on loop 3 of GPHα. To improve assay homogeneity, a single mAb (code: INN-hCG-22) recognizing a broad spectrum of hCG and hCG-like molecules, i.e. hCG, nicked hCG (hCGn), hCG lacking the carboxyl-terminal peptide of the β-subunit (-CTPhCG), hCGβ, hCGβn, -CTPhCGβ and hCGβcf, was labeled with isothiocyanatophenylene triamintetraacetic acid-europium (Wallac, Turku, Finland) according to the manufacturer's recommendations [27] and used as a detection reagent in the 3 assays for hCG, hCGβ and hCGβcf, respectively.

The specificities of the applied IFMAs for hCG and hCG variants are summarized in Table 1.

**Table 1:** Specificity of IFMAs for hCG and/or hCG-variants

Assay type	Assay specificity	Capture mAb Epitope localization	Detection mAb Epitope localization
**hCG**	**hCG **intact αβ heterodimer, bioactive; **hCGn **nicked αβ heterodimer, nicks in the region of aa hCGβ44-48	INN-hCG-45 c_3_ hCGβ loop 2	INN-hCG-22 β_2_ hCGβ loops 1+3 aa 20-25+68-77
**hCGβ**	**hCGβ **intact non-combined free hCGβ-subunit, aa hCGβ1-145; **hCGβcf **core fragment of hCGβ; aa hCGβ6-40 linked to hCGβ55-92; **hCGβn **nicked hCGβ, nicks in the region of aa hCGβ44-48	INN-hCG-68 β_7_ hCGβcf	INN-hCG-22
**hCGβcf**	**hCGβcf-only**	INN-hCG-106 β_11_ hCGβcf	INN-hCG-22
**hCGα**	**hCGα **intact non-combined free a-subunit of hCG; aa hCGα1-92	INN-hCG-72 α_6_ hCGα 33-42	INN-hFSH-158 α_5_ Loop 3 (Tyr 65)

Abbreviations and definitions for hCG and hCG-derived molecules as put forward by the IFCC Working Group for Standardization of hCG (Berger et al., 2002)

aa ... amino acids

### Purification of hCGα from seminal plasma by immunoprecipitation

Seminal plasma samples from three normozoospermic men were pooled and 1 ml of the pool was diluted 1:2 with modified RIPA buffer (10 mM Tris-HCl pH 7.4; 150 mM NaCl; 1% NP-40; 0.25% Na-deoxycholate, complete Mini Protease Inhibitor Cocktail (Roche Diagnostics) and 15 μl monoclonal antibody (4 mg/ml; Code INN-hFSH-132; [18]). The mixture was incubated overnight at 4°C on a rotary shaker. A Protein-G agarose resin (Upstate) was added and samples were incubated for further 2 h at 4°C. Thereafter, samples were centrifuged, supernatant removed and the Protein-G agarose resin was washed 5 times with modified RIPA buffer. The Protein-G agarose resin was heated to 95°C for 10 min in 50 μl modified RIPA buffer and centrifuged at 16,000 g for 10 min. The pellet was discarded and the hCGα-containing supernatant stored at -20°C.

### Digestion with glycosidase

hCGα purified from seminal plasma by IP, as described above (2.4.) and, for comparative purposes, large free hCGα purified by HPLC from supernatant of HEK293 stably transfected with β_2_AR and specifically stimulated with 10 μM isoproterenol [28], as well as the frozen carrier-free concentrate (FC 862) of the WHO adopted 1^st ^International Reference Preparation for Immunoassay of hCGα (1^st ^IRP hCGα 99/720) were deglycosylated with peptide N-glycanase PNGase F (New England BioLabs). For digestion under non-reducing conditions to remove the glycan at Asn^52 ^[29,30], 15 μl samples equivalent to 150 ng hCGα were incubated with 1.5 μl 0.5 M sodium phosphate buffer (pH 7.5), 1.5 μl NP-40 and 1 μl Enzyme (PNGase F, 500 U) for 2 hrs at 37°C. For digestion under reducing conditions to remove both glycan moieties, samples were incubated with 1.5 μl 10× denaturing buffer (5% SDS, 10% β-mercaptoethanol) for 10 min (95°C) and put on ice prior to deglycosylation.

### Western Blot

Samples were diluted with sample buffer to 25 μl, separated via gel electrophoresis on 4% stacking and 13% separating gels and transferred to an Immun-Blot™ polyvinylidene difluoride (PVDF) membrane (Bio-Rad Laboratories). Membranes were probed with mouse monoclonal antibody INN-hFSH-132 at a dilution of 1:2,000 for blots under non-reducing conditions and rabbit hCGα antiserum at a dilution of 1:10,000 for reduced proteins, respectively. Detection was performed with HRP-conjugated secondary antibodies (Promega), chemoluminescent substrate (Amersham ECL™ Western Blotting Analysis System, GE Healthcare) and exposure to ECL Hyperfilm (GE Healthcare).

### Verification of hCGα by mass spectrometry

hCGα purified from seminal plasma by IP, as described above (2.4.), was analyzed by SDS-PAGE, and the protein band at 24 kDa was excised from the gel and digested with endoproteinase Lys-C [EC 3.4.21.50] (Sigma-Aldrich, 1/20 w/w) in 100 mM H_4_HCO_3 _buffer (pH = 8.0) for 2 hours at 37°C. The digest was analyzed using nano-HPLC consisting of an UltiMate 3000 System (Dionex Corporation) connected online to a linear iontrap mass spectrometer ThermoElectron Finnigan LTQ) equipped with a nanospray ionization source. The nanospray voltage was set at 1.6 kV, the heated capillary was held at 200°C.MS/MS, and spectra were searched against a human protein database using SEQUEST (LCQ BioWorks; ThermoFinnigan).

### Statistical analyses

Results are expressed as mean values ± SEM. Statistical differences among groups were calculated by unpaired Student's t-test and considered significant at *P *< 0.05.

## Results

### Characterization of patients: Serum hormone levels

**Figure 2:**
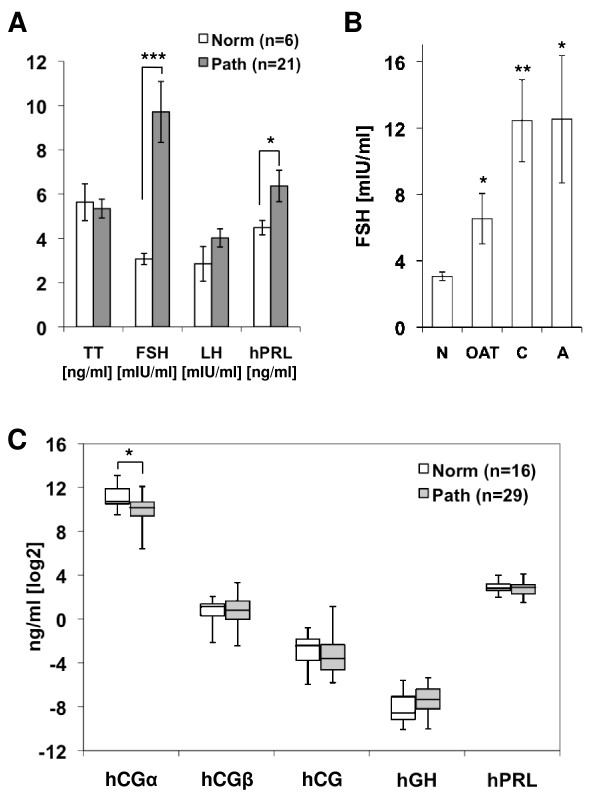
**Serum and seminal plasma hormone levels in men with impaired semen quality**. **(A) **Serum T, FSH, LH and hPRL levels in men with pathologic semen analyses findings (Path) and normozoospermic controls (Norm). Men with impaired semen quality showed significantly elevated FSH and hPRL levels. **(B) **Serum FSH levels were significantly higher in men with oligoasthenoterato- (OAT), crypto- (C) or azoospermia. **(C) **Hormone variants analyzed in seminal plasma revealed significantly lower hCGα levels in patients with abnormal semen analyses compared with controls. Statistical significance was determined by unpaired Student's t-test (* *P *< 0.05; ** *P *< 0.001).

Serum levels of TT, FSH, LH and hPRL were analyzed in 21 patients with abnormal semen analyses (OAT n = 5, asthenozoospermia n = 2, teratozoospermia n = 1, oligozoospermia n = 1, cryptozoospermia n = 8, azoospermia n = 4) and compared with 6 control subjects with normal semen analyses (Figure 2A). Patients with pathologic semen analysis findings showed no significant differences in TT and LH serum levels, while hPRL levels were significantly elevated (6.4 ± 0.7 vs. 4.5 ± 0.3 ng/ml; *P *= 0.02). Mean serum FSH levels were significantly increased in the abnormal group compared with controls (9.7 ± 1.4 vs. 3.1 ± 0.1 mIU/ml; *P *= 0.0001), and elevation of FSH levels (Figure 2B) was more pronounced in patients diagnosed with cryptozoospermia (12.4 ± 2.5 mIU/ml, *P *= 0.003) or azoospermia (12.5 ± 3.8 mIU/ml, *P *= 0.04) than those diagnosed with OAT (6.6 ± 1.5 mIU/ml, *P *= 0.04).

### Decreased hCGα levels in seminal plasma of men with abnormal semen analysis results

Hormone and hormone derivative levels were determined by respective IFMAs in seminal plasma specimens from 29 patients with abnormal semen analyses and compared with samples from 16 control subjects with normal semen analyses (Table 2). hPL and hCGβcf levels were similarly low in both groups, with several samples below the detection limit preventing statistical evaluation. hCGβ, holo-hCG, hGH and hPRL levels were not significantly different in the two cohorts (Figure 2B). However, free hCGα levels were significantly lower in patients with pathologic semen analysis findings (1346 ± 191 vs. 2753 ± 533 ng/ml; *P *= 0.022).

**Table 2:** Hormone and hormone derivative levels [ng/ml] in seminal plasma

Diagnosis	Age [years]		Sperm counts		hCGα		hCGβ		hCGβcf		hCG		hGH		hPL		hPRL	
	range	mean	range	mean	range	mean	range	mean	range	mean	range	mean	range	mean	range	mean	range	mean
N (n = 16)	31-57	37.1 ± 1.7	25.0-87.5	53.7 ± 5.1	726-8754	2753 ± 533	0.23-4.16	2.05 ± 0.29	<0.005-0.010	0.007 ± 0.001	0.016-0.578	0.203 ± 0.040	0.004-0.090	0.023 ± 0.006	<0.005-0.738	0.095 ± 0.072	4.00 ± 16.11	7.82 ± 0.86
Path (n = 29)	21-48	34.1 ± 1.5	0-109.9	n.d.	86-4400	1346 ± 191	0.18-10.00	2.22 ± 0.38	<0.005-0.140	n.d.	<0.02-2.260	0.279 ± 0.101	<0.005-0.086	0.028 ± 0.005	<0.005-1.760	n.d.	2.92-17.57	7.54 ± 0.78
																		
AS (n = 3)	25-42	34.0 ± 4.9	35.6-109.9	77.2 ± 21.9	110-1500	820 ± 402	0.36-10.0	5.29 ± 2.78	<0.05-0.14	n.d.	0.060-2.260	0.840 ± 0.711	<0.005-0.030	n.d.	0.016-1.760	0.629 ± 0.566	n.t./7.50	n.d.
T (n = 1)	42	n.d.	109.3	n.d.	2500	n.d.	2.8	n.d.	<0.005	n.d.	0.82	n.d.	0.007	n.d.	0.21	n.d.	n.d.	n.d.
AST (n = 2)	30	n.d.	n.d/19.5	n.d.	1100-4400	n.d.	2.10-2.16	n.d.	<0.005-0.024	n.d.	0.5-1.3	n.d.	n.t./0.006	n.d.	<0.005-0.160	n.d.	n.t./7.38	n.d.
O (n = 2)	23-45	n.d.	13.1-16.1	n.d.	669-1500	n.d.	0.40-1.02	n.d.	<0.005/n.d.	n.d.	<0.02-0.041	n.d.	<0.005-0.050	n.d.	0.007	n.d.	n.t./3.87	n.d.
OAT (n = 7)	22-36	29.0 ± 1.9	0.6-6.2	3.7 ± 0.8	610-1938	1107 ± 176	0.56-2.00	1.38 ± 0.24	<0.005	<0.005	<0.02-0.234	0.102 ± 0.037	<0.005-0.049	0.029 ± 0.007	<0.02	<0.02	3.34-8.09	5.44 ± 1.16
C (n = 8)	21-45	34.5 ± 2.7	<0.3-0.3	n.d.	192-3661	1522 ± 428	0.70-3.39	2.04 ± 0.41	<0.005	<0.005	<0.02-0.420	0.122 ± 0.016	<0.005-0.086	0.039 ± 0.012	<0.02	<0.02	2.92-17.57	8.85 ± 1.73
A (n = 6)	31-48	39.8 ± 3.3	0	0	86-2630	1108 ± 394	0.18-5.21	2.35 ± 0.82	<0.005	<0.005	<0.02-0.120	0.066 ± 0.018	0.003-0.037	0.016 ± 0.006	<0.02	<0.02	7.00-8.86	7.88 ± 0.34

Patients were grouped according to spermiogram (WHO 1995): normozoospermia (N), abnormal semen analysis results (Path), asthenozoospermia (AS), teratozoospermia (T), asthenoteratozoospermia (AST), oligozoospermia (O), oligoastheoteratozoospermia (OAT), cryptozoospermia (C), azoospermia

### Decreased seminal plasma holo-hCG levels in patients with decreased sperm count

**Figure 3:**
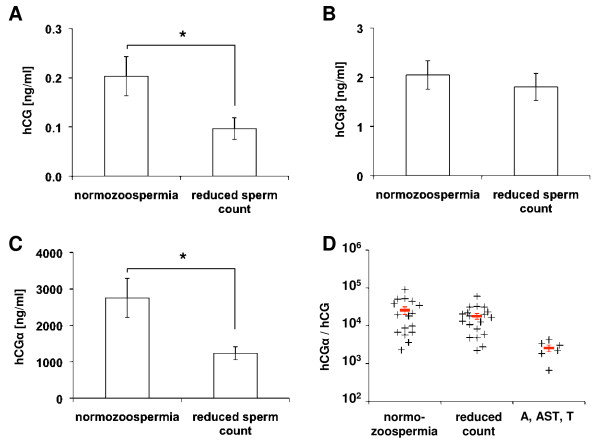
**Reduced hCG and free hCGα seminal plasma levels in men with reduced sperm count**. **(A) **Patients with reduced sperm count (n = 23) showed significantly lower free holo-hCG levels compared with normozoospermic controls (n = 16), while **(B) **free hCGβ levels were not affected. **(C) **Similar to holo-hCG, free hCGα levels were significantly lower in men with reduced sperm count, resulting in **(D) **hCGα/hCG ratios comparable to normozoospermic men, while men with asthenozoospermia, teratozoospermia or asthenoteratozoospermia (AS, T, AST; n = 6) showed reduced hCGα/hCG ratios. Red bars indicated mean ± SEM. **(A-C) **Statistical significance was calculated by Student's t-test (* *P *< 0.05).

In patients with impaired semen quality, the highest variability of all analyzed hormones was observed for holo-hCG (0.279 ± 0.101 ng/ml, Table 2). Patients with astheno-, terato- or asthenoteratozoospermia had higher, but statistically insignificant, hCG seminal plasma levels. On the other hand, patients diagnosed with reduced sperm counts (oligo-, oligoasthenoterato-, crypto- or azoospermia; n = 23) had significantly reduced hCG levels compared with normozoospermic men (0.097 ± 0.022 vs. 0.203 ± 0.040 ng/ml, *P *= 0.028, Figure 3A). While hCGβ levels were not significantly different (1.80 ± 0.27 vs. 2.05 ± 0.29 ng/ml, *P *= 0.55, Figure 3B), mean hCGα levels were significantly reduced in patients with reduced sperm counts (1237 ± 197 vs. 2753 ± 533 ng/ml, *P *= 0.015, Figure 3C). Given the fact that hCG as well as hCGα were similarily reduced by approximately 50-60% in both cohorts, the ratio of hCGα/hCG was analyzed and found to be comparable (normozoospermia: 25.7 ± 6.1 × 10^3^, reduced sperm count: 17.9 ± 3.2 × 10^3^, Figure 3D). Patients with astheno-, terato- or asthenoteratozoospermia had a strongly reduced hCGα/hCG ratio (2.6 ± 0.5 × 10^3^, Figure 3D).

### Genuine large hCGα in seminal plasma

Given the high free hCGα levels in seminal fluid compared with serum (approximately 10,000-fold higher, as described previously [13]) and the decreased levels in seminal plasma of patients with poor semen analysis, the hormone derivative from these patients was purified and investigated in more detail.

**Figure 4:**
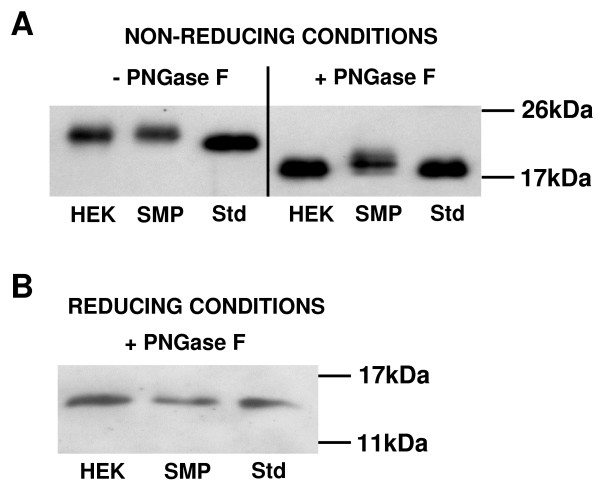
**hCGα purified from seminal plasma differs in glycosylation from both HEK293-derived large hCGα and hCGα dissociated from pregnancy-derived intact hCG**. **(A) **Seminal plasma-derived hCGα (SMP) had an M_r,app _of 24 kDa similar to the large free subunit purified from supernatant of HEK293 cells stably transfected with β_2_AR and stimulated with isoproterenol (HEK), whereas the pregnancy-derived dissociated subunit (Std; 1^st ^IRP hCGα 99/720) had an apparent molecular mass (M_r,app_) of 22 kDa. Digestion with PNGase F under non-reducing conditions (deglycosylation of Asn^52^) resulted in monoglycosyl hCGα subunits of identical size for HEK and Std (M_r,app _= 18 kDa), while SMP migrated with an M_r,app _of 19 - 20 kDa. **(B) **After digestion with PNGase F under denaturing conditions (deglycosylation of both N-glycosylation sites Asn^52 ^and Asn^78^), hCGα from all three sources migrated identically (Mr,app = 15kDa), indicating identical amino acid backbones.

Under non-reducing conditions, hCGα dissociated from pregnancy-derived holo-hCG migrated as an approximately 22 kDa band (Figure 4A). Large free hCGα, which is unable to associate with β-subunits due to larger N-linked sugar chains [31], had an apparent molecular mass (M_r,app_) of 24 kDa. hCGα isolated form seminal plasma migrated comparable with HEK293-derived large free hCGα. After digestion of the glycan at Asn^52^, the dissociated standard and HEK293-derived large free hCGα showed identical M_r,app _of 18 kDa, while protein isolated from seminal fluid migrated as a diffuse band of approximately 19 - 20 kDa (Figure 4A). When both glycan moieties were removed, all three hCGα variants tested resulted a 15 kDa deglycosylated protein.

The biochemical identity of hCGα purified from seminal fluid was verify by nano-HPLC MS/MS analysis after digestion with endoproteinase Lys-C (Table 3).

**Table 3:** MS/MS analysis of free hCGα isolated from seminal plasma

Sequence	MH+	% Mass	Position	% AA
TMLVQK	719.41	5.51	70 - 75	5.17
SYNRVTVMGGFK	1358.41	10.40	88 - 99	10.34
Total	2059.08	15.76	18	15.52

The identified amino acid (AA) sequences after Lys-C digestion, the 1+ charge state [MH+] of the hCGα protein fragments, the percent mass, the positions of amino acid residues, and percentage of the amino acid sequence obtained by database search using SEQUEST are shown.

## Discussion

Protein and glycoprotein hormone-like substances have been described previously in human seminal fluid, but the lack of antibodies and consequent sandwich assays with clearly defined glycoprotein hormone variant recognition patterns meant that a general variant profile of these markers and their relationship to fertility disturbances could not be accomplished. We analyzed the profile of endocrine parameters in patients with abnormal semen analysis findings in comparison to normozoospermic men to elucidate a putative pathophysiological role of glycoprotein hormone variants in spermatogenesis disorders.

To characterize the patient cohort serum levels of TT, FSH, LH and hPRL were analyzed. In accordance with previous reports [32], significantly higher serum hPRL levels were found in men with abnormal semen analysis, but still within the normal range, indicating that the patients did not suffer from hyperprolactinemia. Mean serum FSH levels were above the normal reference range (1.3 - 8.4 mIU/ml [33]) in patients with abnormal semen analysis and highest in patients with cryptozoospermia and azoospermia, reflecting a decline in function of seminiferous tubules [34]. High heterogeneity of FSH levels indicated that men with both obstructive and non-obstructive azoospermia were included in the study cohort.

In seminal plasma levels of hCGα were found to be significantly lower in patients with abnormal semen analyses. Similarly, in patients with reduced sperm count, intact hCG levels were significantly lower. In these patients, the hCGα/hCG ratio was comparable with normozoospermic men, indicating co-secretion of free hCGα and hCGα associated with hCGβ. Compared with hCG, free hCGβ is present in seminal plasma in an approximately 10-fold, and free hCGα in an approximately 10,000-fold excess. Thus, free hCGα appeared as large subunit, distinct from the α subunit present in hCG [31] and unable to associate with the available β subunit. Non-associable and associated hCGα seem to be secreted at a constant ratio of approximately 10,000:1, the latter being the rate limiting subunit of holo-hCG association. On the other hand, the hCGα/hCG ratio was reduced in patients with astheno-, terato- or asthenoteratozoospermia, although the limited number of patients in this cohort require confirmation of these alterations by further research. Other hormone variants were not significantly different in the cohorts tested and generally expressed at low levels.

The pathophysiological role of reduced hCGα and hCG levels is uncertain. We previously showed hCGβ expression in the testis in peritubular and presumably Leydig cells [13]. Due to its structural homology to LH, hCG is able to stimulate T production by Leydig cells. Thus, it could be hypothesized that hCG expression in the testis serves as a local backup system to sustain basal T secretion. However, hCG levels in seminal plasma were very low (0.016 - 0.578 ng/ml in normozoospermic men). During pregnancy, the serum ratios of hCG to the free α and β subunits is usually in the range in 100:1, while in seminal plasma, hCGα is found in 10,000-fold excess (726 - 8754 ng/ml in normozoospermic men) of the holo-hormone.

The molecular function of the free hCGα is still unresolved, and no receptors for the free subunit have yet been described. Free hCGα has been reported to stimulate endometrial stromal cell differentiation synergistically with progesterone [35]. Antisense hCGα RNA reduced the tumorigenic potential of lung cancer cells [36] and hCGα inhibited growth of prostatic stromal cells [37]. In the male reproductive tract, several sources of free hCGα have been described, in particular the prostate [37-39]. Moreover, significant amounts of hCGα have been detected in seminal vesicle fluid, and minor concentrations in the testis [13,40]. Herein, we demonstrate that hCGα purified from seminal plasma had the M_r,app _of large free hCGα, which is incapable of associating with β-subunits due to larger N-linked sugar chains [31]. Interestingly, the large free α subunit isolated from seminal fluid showed a different glycosylation pattern than large hCGα produced from HEK cells stably transfected with β_2_AR and stimulated with isoproterenol [28]. While the latter appeared to be more highly glycosylated at Asn^52^, seminal plasma-derived hCGα appeared to be more highly glycosylated at Asn^78^. This might reflect distinct molecular functions of large free hCGα variants from different sources due to alternative glycosylation. After total deglycosylation, hCGα derived from seminal plasma, HEK cells and dissociated from the heterodimeric hCG showed identical M_r,app_, indicating identical amino acid backbone and genuineness of large free hCGα in seminal plasma as shown by nanospray MS. Thus, due to its unique gylcosylation pattern, free hCGα purified from seminal plasma, where it is normally present in high concentrations and is reduced in men with abnormal semen analysis, represents a promising variant to investigate the unresolved molecular function of the free subunit.

## Conclusions

Levels of free hCGα, by far the most abundant hCG variant in seminal plasma, were significantly reduced in men with abnormal semen analyses. Additionally, in men with reduced sperm counts, holo-hCG levels were accordingly lower, indicating a pathophysiological relevance of these hormone variants in spermatogenesis. hCGα in seminal plasma was identified as being a highly glycosylated large free subunit with a unique glycosylation pattern. Alternative glycosylation clearly might modify the function of the hormone subunit, thus free large hCGα purified from seminal plasma represents a promising molecular entity to further investigate the physiological role of free hCGα in spermatogenesis.

## Competing interests

The authors declare that they have no competing interests.

## Authors' contributions

CZ performed the statistical analysis and drafted the manuscript. RG carried out the immunoassays. MG and EP participated in the design of the study and determined serum hormone levels and sperm parameters. HL performed mass spectrometry. PB conceived of the study, participated in its design and coordination and helped to draft the manuscript. All authors read and approved the final manuscript.
